# Tumefactive Multiple Sclerosis in Taiwan

**DOI:** 10.1371/journal.pone.0069919

**Published:** 2013-07-18

**Authors:** Yi-Chun Kuan, Kai-Chen Wang, Wei-Hsin Yuan, Ching-Piao Tsai

**Affiliations:** 1 Department of Neurology, Shuang-Ho Hospital, Taipei Medical University, Taipei, Taiwan; 2 Neurological Institute, Department of Neurology, Taipei Veterans General Hospital, Taipei, Taiwan; 3 National Yang-Ming University, Taipei, Taiwan; 4 Department of Neurology, Cheng Hsin General Hospital, Taipei, Taiwan; 5 Division of Radiology, Taipei Municipal Gan-Dau Hospital-T.V.G.H., Taipei, Taiwan; 6 Department of Radiology, Taipei Veterans General Hospital, Taipei, Taiwan; National Institutes of Health, United States of America

## Abstract

**Background:**

Multiple sclerosis (MS) is less common in Asia, including Taiwan, and some characteristics of MS in Asians differ from those of Caucasians. Tumefactive brain lesion is even rarer in MS patients.

**Objective:**

To review patients with tumefactive MS and compare them with those in other studies investigating tumefactive demyelinating lesions and our Taiwanese typical MS patients.

**Methods:**

Twelve patients (6.3%) from the 190 MS patients visiting Taipei Veterans General Hospital from 1985 to 2010 were enrolled. They all fulfilled the McDonald or Poser criteria for MS and had at least one brain lesion larger than 2 centimeters with or without a mass effect.

**Results:**

Eleven patients (91.7%) were female and presented tumefactive brain lesions during the first attack. The clinical course of all patients was relapsing-remitting with a second attack within 2 years. Their initial extended disability status score was higher, but the prognosis was better after more than 2 years of follow-up, than in other studies. Moreover, our patients did not have optic or spinal involvement as well as positive neuromyelitis optica immunoglobulin or aquaporin-4 antibody, which is very common in Taiwanese MS patients.

**Conclusion:**

Tumefactive MS is not common in Taiwan. Although the tumefactive demyelinating lesions seem to be terrible initially, their prognosis is relatively more favorable than expected.

## Introduction

Multiple sclerosis (MS) is a chronic inflammatory demyelinating disease of the central nervous system (CNS). It is far more common in Western countries, with a prevalence of >50 per 100,000 population, much higher than that in Asia [1]. In Taiwan, a 1976 study reported an estimated regional prevalence in northern Taiwan of 0.8 per 100,000 [2], and another study in 2004 using the registration data of the Bureau of National Health Insurance reported a prevalence of 1.9 per 100,000, similar to other Asian series [3]. The characteristics of MS in Taiwan are different from those of Caucasians; for example, there is more optic and spinal involvement, relatively rapid progression, and a lower frequency of oligoclonal bands (OBs) and raised IgG index in the cerebrospinal fluid (CSF) [2]–[4].

The brain lesions of MS are typically multiple, small, 3 to 15 mm in diameter, and round or ovoid without a mass effect [5]. They are usually located in the periventricular white matter, oriented perpendicularly to the long axis of the lateral ventricle. Other locations, such as the corpus callosum, centrum semiovale, juxtacortical regions, pons, floor of the fourth ventricle, cerebellar peduncles, or cerebellar hemispheres, are also often observed on T2-weighted imaging [5]. However, atypical imaging features with larger lesions in the cerebral hemispheres resembling brain tumors have been described. This is referred to as a tumefactive demyelinating lesion (TDL), which is characterized by a brain lesion, 2 cm or more in diameter, associated with or without a mass effect, perilesional edema or the presence of ring enhancement on neuroimaging [6], [7]. The occurrence of TDL is uncommon, and is estimated at 1–2/1000 cases of MS [8]. The prevalence has been suggested as 3 cases per million inhabitants per year [9]. Although TDL case reports have been published [6], [7], there have been fewer among Asians [10]–[12], and no Taiwanese study so far.

The purpose of this study was to investigate the clinical presentations, radiological features, laboratory and pathological findings, treatment and prognosis of tumefactive MS in Taiwan, and to compare this with typical MS in Taiwan and previous studies of tumefactive MS in other countries.

## Materials and Methods

We reviewed the brain MRI of 190 patients with MS who had visited Taipei Veterans General Hospital, a tertiary referral medical center located in northern Taiwan, from January 1985 to December 2010. We enrolled patients who had fulfilled either McDonald or Poser criteria for MS [13], [14] at the last follow-up and whose brain imaging showed at least one lesion more than 2 centimeters in size with or without a mass effect. Those with a prior history of concomitant neoplasm, infection, vascular lesion, or brain irradiation were excluded.

By medical record review, the clinical data, including gender, date of birth, age at first onset, mean duration of relapse, neurological symptoms and signs (cognition, seizure, visual acuity, visual field, eye movement, motor, sensory, brainstem, cerebellar, involuntary movement, and autonomic) during attacks, clinical course, number of attacks, magnetic resonance imaging (MRI) of the brain including the spine or not, laboratory findings of CSF and biochemistry including IgG index and OBs, aquaporin-4 (AQP4) antibodies analyzed at our laboratory and/or neuromyelitis optica immunoglobulin (NMO-IgG) at the Mayo Clinic, evoked potentials, pathology of the brain lesion if available, treatment, and functional outcome assessed according to the extended disability status score (EDSS), were collected and analyzed. All the clinical information was obtained by board-certified neurologists and radiologists, and the pathologies were also verified by board-certified pathologists.

Brain lesions seen on MRI were graded by size as either small (≤2 cm), middle (2–5 cm) or large (>5 cm), and spinal lesions as short cord (<3 segments) or long cord (≥3 segments) involvement.

This study was approved by the institutional review board (No. 2013-04-033BC) of Taipei Veterans General Hospital. The patient records were anonymized during the processing of data analyzing and no authors had direct contact with the patients.

## Results

From our database of 190 MS patients, we identified 12 (6.3%) who fulfilled our inclusion criteria. The general characteristics and clinical neurologic symptoms and signs of these patients are shown in Table 1. They were predominantly female (11/12 = 91.7%) and the median age at the first attack was 41.5 years (16–62). The most common initial neurological presentation was numbness (83.3%), followed by weakness (50%), cognitive impairment (33.3%), brainstem symptoms/signs such as double vision (25%), visual field defects (16.7%), and incoordination (8.3%). Some patients developed seizure, optic neuritis and/or urine/stool/sexual dysfunction during the following episodes. All of them had suffered from focal limbs and/or facial weakness, but none had involuntary movement. All of them had relapsing-remitting MS and the second attack occurred within 2 years (mostly within one year) after the first attack. Most (11/12 = 92%) of their brain images showed lesion(s) larger than 2 cm during the first attack and 3 (25%) developed another tumefactive brain lesion during the following attack.

**Table 1:** General characteristics and clinical neurologic symptoms and signs.

No#	Sex	Onset (y/o)	Course	Times^a^	Motor	Mental	Sensory	Cb	BS	VF	Sz	ON	ANS	EPS	Initial EDSS	Follow (years)	Current EDSS
1	F	62	RR	2	1,2 b	0	1,2	2	0	0	0	0	0	0	5.5	3	3.5
2	F	17	RR	2	2	0	1,2	0	1	0	0	0	0	0	4.5	3	0
3	M	55	RR	2	1	1	1	0	0	0	2	0	0	0	5.0	3	0
4	F	49	RR	2	1	1,2	1,2	0	0	1	0	0	0	0	5.5	5	2.5
5	F	41	RR	6	1,4	1	1,3,4	0	1,2,5	0	0	3	0	0	4.5	7	8.5
6	F	42	RR	2	1,2	0	1,2	1	0	1	0	0	2	0	6.0	5	5.5
7	F	39	RR	2	2	2	0	0	0	0	0	0	0	0	3.5	4	0
8	F	21	RR	2	2	0	1	2	2	0	0	0	0	0	3.0	2	1.0
9	F	44	RR	2	2	1,2	0	0	0	0	0	0	0	0	6.0	10	3.5
10	F	27	RR	2	2	0	1	0	0	0	0	0	0	0	3.0	3	0.5
11	F	54	RR	10	1,2,3,4,6,7,8,9,10	5,8	1,6,9,10	0	2	0	0	3	4	0	7.0	15	6.0
12	F	16	RR	5	2,3	2	1,2,3,4,5	0	1,2	2	0	0	0	0	2.5	8	0
															4.7 c		2.6 c

Motor: focal limbs or facial weakness; Mental: impaired cognition; Sensory: hypsthesia, hyperesthesia; Cb: cerebellar dysfunction-incoordination; BS: brainstem sign-diplopia, bulbar sign; VF: visual field defect; Sz: seizure; ON: optic neuritis; ANS: autonomic-orthostatic hypotension, urine/stool/sexual dysfunction; EPS: extrapyramidal tract-involuntary movement; EDSS: extended disability status score; RR: relapsing-remitting.

a Times of attack.

b The number means the neurological symptoms and signs during the time of attack.

c Average EDSS.

**Figure 1:**
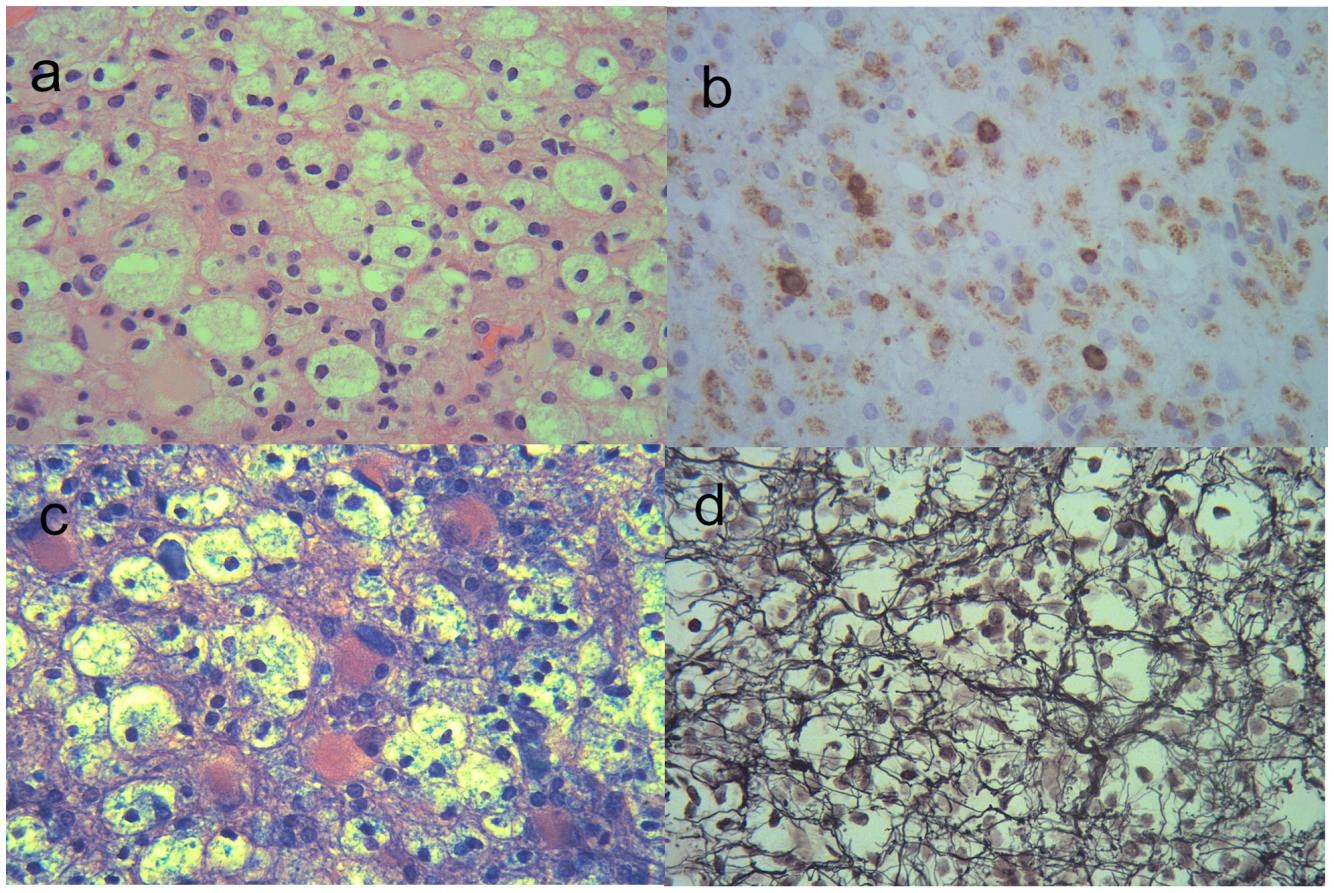
Pathology of a biopsied tumefactive brain lesion (patient #4). It shows some characteristic features of demyelination include hypercellularity and reactive gliosis (a: haematoxylin-eosin), abundant foamy macrophages (b: CD 68 antibody; for macrophage), myelin loss (c: Luxol-fast blue; for myelin), and “relative” axonal preservation (d: Bodian; for nerve fibers).

**Figure 2:**
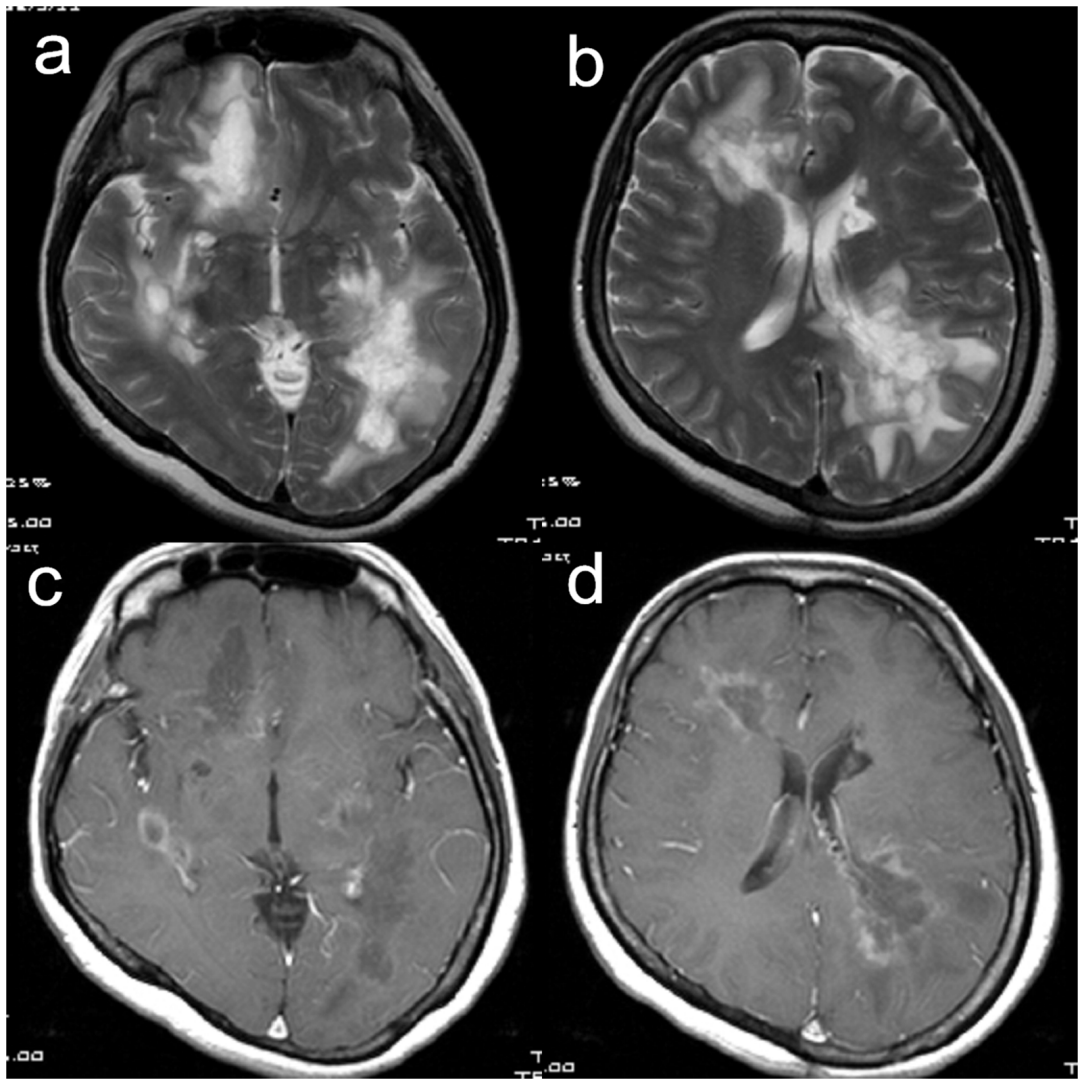
T2-weighted magnetic resonance images (a, b) of the patient #4. They show multiple large hyperintensity lesions over periventricular and juxtacortical area of right frontal and left parietal lobes with perifocal edema. Thalamus, basal ganglion and internal capsule are also involved. Gadolinium-enhanced T1-weighted (c, d) images of the patient also show ring and heterogenous enhancement in right frontal and left parietal lobes.

**Figure 3:**
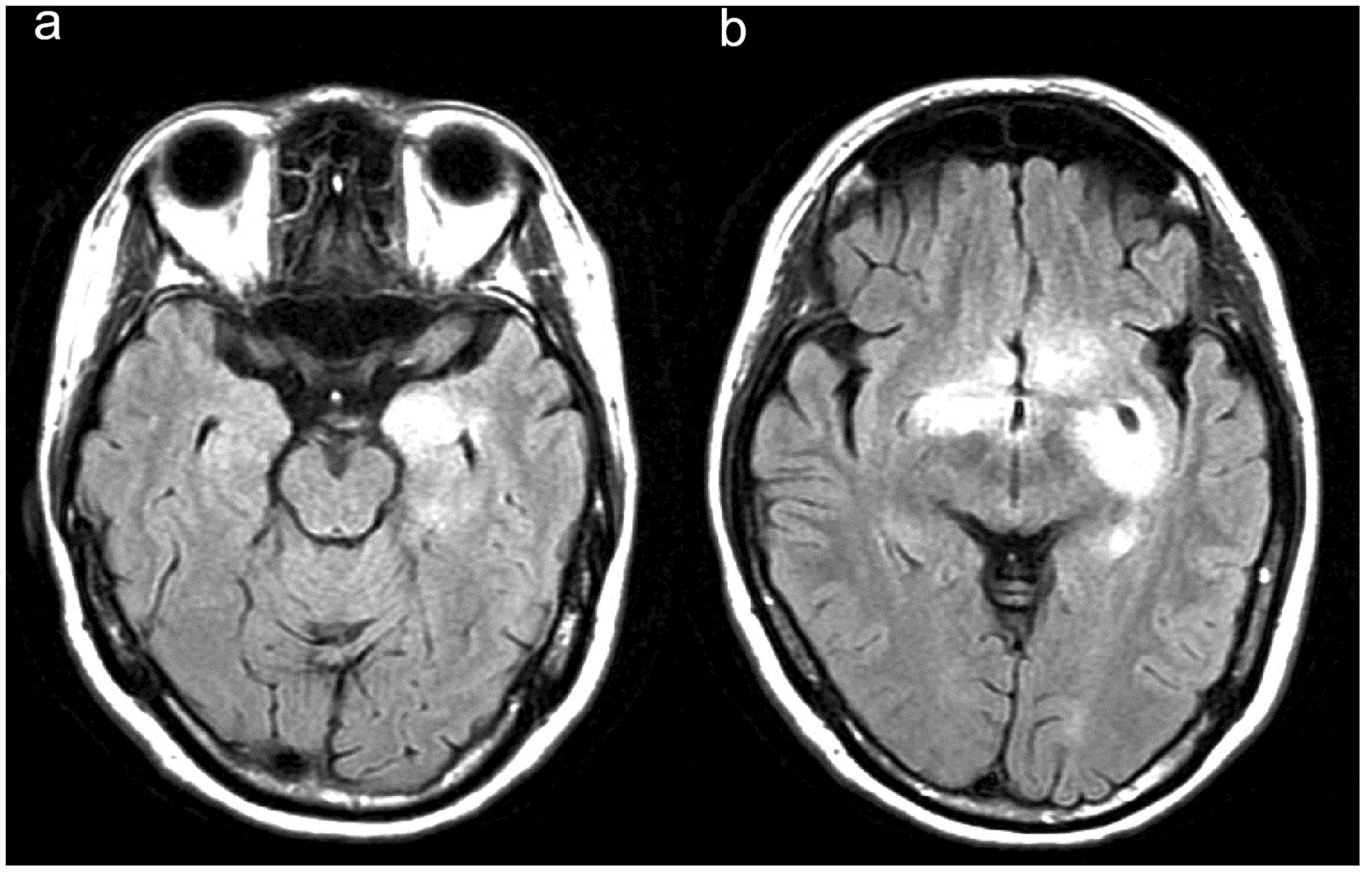
Fluid attenuated inversion recovery (FLAIR) magnetic resonance images (a, b) of another patient #7. They show multiple hyperintensity lesions over juxtacortical area of left medial temporal lobe and bilateral basal ganglion (caudate nucleus and putamen). Pons and cerebellum are also involved.

The MRI results of the brain and/or spine are summarized in Table 2. Four patients with large (>5 cm) brain lesions had undergone brain biopsy for the tumor-like lesion. The pathologies revealed a demyelinating process (hypercellularity, myelin loss, abundant foamy macrophages, and “relative” axonal preservation) without neoplasia (Figure 1). Nine patients (75%) had enhancement (8 rings, 3 heterogeneous, 2 homogeneous, and 1 nodular pattern) in the tumefactive lesions and 6 patients (50%) had more than 2 foci of the tumefactive lesions (Figure 2 and 3) during the first attack. Periventricular (50%) and juxtacortical (50%) regions were mostly affected by the tumefactive lesions. Five (41.7%) patients also had abnormal findings on the cervical and/or thoracic spinal MRI.

**Table 2:** Magnetic resonance image findings (location, size and enhancement).

No	PV	JC	Thalamus	IC/BG	Midbrain	Pons	Medulla	Cerebellum	Splenium	Hypothalamus	Spinal cord
1	L * a	S *	–	–	–	–	–	–	S *	–	ND
2	L*	S*	S *	S*	S*	M *	S*	–	–	–	ND
3	–	L * ,M *,M	–	–	–	–	–	–	–	–	ND
4	L *	M *	S	S	–	–	–	–	–	–	ND
5	S	–	–	–	–	M	S	S	S	–	Ts*
6	S	S	M *	S	S	S	–	S	–	S	Cs*,Tl*
7	–	M	–	M *,S	–	S	–	S	–	–	ND
8	–	M *,M	–	–	–	–	–	–	–	–	Cs*
9	S	L,M	–	–	–	–	–	–	–	–	ND
10	M	S	–	–	–	–	–	–	–	–	Ts
11^b^	M	M*,M*	S	S	S	S*	S	–	S	–	Cs * , Tl *
12	M * ,M * ,M *	S *	S	S	–	S *	S*	–	M *	–	ND

PV: periventricular white matter; JC: juxtacortical; IC: internal capsule; BG: basal ganglion (caudate, putamen, globus pallidum).

S: small, ≤2 cm; M: middle, 2–5 cm; L: large, >5 cm;

* enhancement.

ND: not done; C: cervical cord; T: thoracic cord; s: short, <3 segments; l: long, ≥3 segments.

a lesions during the first attack are underlined.

b Only spinal lesion without brain lesion noted during the first attack; her large brain lesion was noted during the second attack.

All patients received lumbar puncture for CSF study. The mean protein level was 43.3 mg/dl (normal limit: 15–45 mg/dl; range 21–71 mg/dl) and the white blood cell count/mm^3^ was 7.0 (normal limit: <5; range 0–26). IgG index was higher than 0.67 in 5 (50%) of 10 patients. OBs were examined in 4 patients and 3 were positive (75%). Eleven of 12 patients (92%) underwent either NMO-IgG and/or AQP4 antibodies analysis, and all were negative. Six patients received a visual evoked potential (VEP) study and 2 (33.3%) had prolonged P100 latency (Table 3).

**Table 3:** Laboratory examinations and treatment.

No	CSF protein	CSF sugar	CSFWBC	CSFRBC	IgG index	OB	NMO-IgG	AQP4 Ab	EP	Pathology	Steroid	Disease modifying drugs	Immuno-suppressant
1	39	72	0	4	0.47	ND	negative	negative	ND	demyelinating	yes	INF	azathioprine
2	40	78	2	0	0.62	ND	ND	negative	VEP: normal	demyelinating	yes	INF	–
3	55	NA	1	1	0.36	ND	ND	ND	VEP: normalBAEP: normalSSEP: right abnormal	demyelinating	yes	INF	–
4	30	68	1	24	0.80	ND	ND	negative	ND	demyelinating	yes	INF	–
5	44	64	19	NA	0.92	positive	negative	negative	VEP: left abnormal	ND	yes	glatiramer acetate, INF	azathioprine, mitoxantrone
6	53	52	26	0	NA	normal	ND	negative	ND	ND	yes	INF	–
7	38	75	13	NA	0.75	ND	ND	negative	VEP: normalSSEP: normal	ND	yes	INF	–
8	31	60	0	82	0.89	ND	ND	negative	ND	ND	yes	–	cladribine
9	50	71	2	1	0.42	ND	ND	negative	NA	ND	yes	–	–
10	21	73	1	0	0.82	ND	ND	negative	ND	ND	yes	–	–
11	71	62	8	12	NA	positive	ND	negative	VEP: bilateral abnormal	ND	yes		azathioprine
12	48	69	11	NA	1.02	positive	ND	negative	VEP: normal	ND	yes	INF	–

CSF: cerebrospinal fluid; OB: oligoclonal band, NMO-IgG: neuromyelitis optica immunoglobulin; AQP4 Ab: aquaporin-4 antibody; EP: evoked potential; VEP: visual EP; BAEP: brainstem auditory EP; SSEP: somatosensory EP; INF: β-interferon; ND: not done; NA: not available.

All 12 patients received steroid therapy (oral and pulse) shortly after the attack, with clinical and/or radiographic improvement. Beta-interferon was administered to 9 (75%) patients, and 5 (41.7%) were given an immunosuppressant such as azathioprine, mitoxantrone or cladribine thereafter (Table 3). The mean EDSS during the first attack and the last follow-up (at least 2 years) was 4.7 (range 2.5–6.0) and 2.6 (range 0–8.5), respectively (Table 1). Four patients achieved total recovery and 2 had only mild functional impairment (EDSS less than 1) of the hand after more than 2 years of follow-up.

## Discussion

This is currently the largest report on TDL in Taiwan, including 12 patients with tumefactive MS from our 190 MS patients (6.3%), which is a higher proportion than the previous estimation of 0.1–0.2% of MS patients [8]. However, the higher percentage may not represent the actual prevalence of tumefactive MS in Taiwan, because the study included only those patients visiting a tertiary medical center in Taipei City, where more complicated cases, such as tumor-like brain lesions, would be referred.

Our patients with tumefactive MS were almost all female (91.7%). Although MS usually occurs in female patients, this is a much higher proportion than in other studies investigating TDL [6], [10], [11], [15] and in Taiwanese typical MS studies [2]–[4]. The age at onset of our cohort was similar to that of other studies, but the median age at onset was a little older. For the clinical course of MS, all of our patients were relapsing-remitting, in contrast to only half of a large cohort of Caucasian tumefactive MS patient [6] and in a Taiwanese typical MS study (Table 4) [3].

**Table 4:** Comparison of studies regarding tumefactive demyelination lesions and a Taiwanese MS study.

Study	Taiwan, 2004 [3]	Taiwan (this study)	USA and Germany,2008 [6]	USA, 1996 [7]	India, 2010 [12]	Japan, 2011 [10]	China, 2009 [11]
Inclusion criteria	Poser’s criteria of MS	Tumefactive (≥2 cm onT2WI) MS	CNS IDD by biopsy	demyelinating (biopsy)lesion ≥2 cm	TDL as first event	TDL≥3 cm on T2WI	TDL by biopsy (spinal lesion not included here)
Total number	45	12 (4 biopsied)	168 biopsied	21 biopsied	14 (8 biopsied)	12 from 102 MS(4 biopsied)	6 (not include 3 spine)
Gender (F:M)	5	11	1.2	1.5	1	1	1
Age of onset(median)	30 (11–70)	41.5 (16–62)	37 (8–69)	37 (10–58)	30 (8–61)	27 (17–64)	29.5(4–45)
Course	51% RR, 30% SP, 19% PP	100% RR	51% RR, 24% MP, 11% SP	Not sure MS	Not sure MS	58% RR, 25% MP, 17% SP	not follow to MS
EDSS and durationof follow-up	64.8% no∼mild;10 years	4.7→ 2.6 (50% 0–1);>2 years	3.5 (3,4.5) →3 (1.5,4)	NA	5.93 →1.75;>2 years	3.5 (1–9.5) → (1–9),67% decreased;60 (2.6–169) weeks	NA
Initial presentation	motor (88%), sensory(84%), visual (58%)	numb (83.3%),motor (50%)	motor (50%), cognitive(43%), sensory (36%)	motor (28.6%), visual(23.8%), cognition (19%)	motor (79%),cognitive (43%)	Motor (75%), visual(58%), AMS(33%),headache (33%)	motor (83%), seizure (33%)
Multiple brainlesions	NA	92%(50% multiple large)	70% (pre-biopsy), 83%(post-biopsy)	52%	50%	100% (17% 2 large)	33%
Size	NA	≥2 cm	4 (0.5–12 cm)	2–11 cm	3.8 (2.5–7.2) cm	≥3 cm	≥2 cm
Location	47% SC, 33% opticneuritis, 9.3%cerebrum, 6.9%cerebellum, 4.7%BS	50% PV, 50% JC	biopsied: 50% F, 42% P;non-biopsied: 79% PV,61% JC, 54.5% SC	F = FP (40%)>O (20%)	93% supratentorial (F>P),3 additional infratentorial, 1 only BS	F (43%), P (14%),O (14%), T (29%)	67% hemisphere, 17% pons, 17% BG
Spinal lesions	46.5% spinal cordlesion	41.7%	38% of 24 (pre-biopsy)	NA	0	41.70%	NA
NMO-IgG	NA	0 of 11	NA	NA	NA	17% of 6	NA
Pathology	NA	demyelination	CNS IDD	NA	8 IDD, relative axonalsparing	inflammatorydemyelinating	TDL

MS: multiple sclerosis; T2WI: T2-weighted image; CNS IDD: central neural system inflammatory demyelinating disease; TDL: tumefactive demyelinating lesion; RR: relapsing-remitting; SP: secondary progressive; PP: primary progressive; MP: monophasic; EDSS: extended disability status score; NA: not available; AMS: altered mental status; SC: spinal cord; BS: brainstem; PV: periventricular white matter; JC: juxtacortical; F: frontal; P: parietal; O: occipital; T: temporal; BG: basal ganglion; NMO-IgG: neuromyelitis optica immunoglobulin.

The most common presentations during the first attack in our study were sensory deficits (83.3%), motor weakness (50%) and cognitive impairment (33.3%), which was similar to most other studies of TDLs (Table 4) [6], [7], [10]–[12]. Sensory and motor dysfunction was also common in typical MS, but cognitive impairment occurred less frequently [3]. This may be explained by the size of the brain lesions, in that a larger lesion would more likely influence cognition or consciousness.

Asians have been reported to have a higher frequency of an optico-spinal form of MS than other groups [16]. The percentage of visual impairment at the onset of illness in Taiwanese typical MS was about 33% [3], which was midway between that of a Japanese (43%) [17] and a Western series (13–28%) [18]. However, visual impairment was found less often (14–16.7%) in our and other Asian studies of TDLs [11], [12], except a Japanese study with 58% [10].

Our patients (50%) had multiple tumefactive brain lesions in the initial brain MRI. Most of the lesions were located in the supratentorial (>80%), especially the periventricular and juxtacortical area, and more than half of them had enhancement. Around 40% of our patients also had spinal lesions; the rate was higher than among Western patients with TDL [6], but lower than among Taiwanese with typical MS (Table 4) [3].

The percentages of elevated IgG indices and abnormal VEP were similar to that of larger tumefactive studies [6] and typical MS [3] in Taiwan –35–50% and 33–37.5%, respectively. However, the percentage of present OBs was higher in our study, and none of the patients had abnormal brainstem auditory evoked potential (BAEP). Although NMO-IgG/AQP4 antibody may also be presented in cases with MS [19], we found neither NMO-IgG nor AQP4 antibody positive cases in our cohort with TDLs.

All of our patients had received steroid and some of them also received an additional immunosuppressant or modifying agent. Compared to other studies, and in spite of the large tumefactive brain lesions at onset and severer initial dysfunction (higher EDSS), the prognosis of our patients was not necessarily severe and even a little more favorable (Table 4). Half of our patients achieved almost total recovery or only mild residual functional impairment (EDSS less than 1) after follow-up for more than 2 years. The size and location of the lesions were not associated with the clinical course, as in other reports [6], [10].

The study reported in the present paper has certain shortcomings. First, the numbers of the cohort of tumefactive MS are rather small. Second, the size of 2 cm, which is used in most published articles regarding tumefactive lesions [6], [7], seems to be a relatively generous (small) selection criterion for tumefactive brain lesions, so our group is a little heterogeneous and some selection bias presents. Third, the duration of follow-up is relatively short. We may overstate the different characteristics to other cohorts and the slightly favorable prognosis. In order to obtain more reliable data, future work is needed to continue tracking the outcome of the cohort and investigate more patients with tumefactive brain lesions. It may also be necessary to further determine the relationship between tumefactive MS, NMO-IgG or AQP4 antibodies and NMO.

### Conclusion

Tumefactive brain lesion is an unusual MS pattern in Western countries as well as in Asia, including Taiwan. Although there were different inclusion criteria between studies, our patients had some distinct characteristics, such as being predominantly female and having a present tumefactive brain lesion during the first attack, all having a relapsing-remitting course with a second attack within 2 years, and higher EDSS initially but a better prognosis after more than 2 years of follow-up. Some characteristics were similar to those of other studies, including the predominantly middle-age onset, more weakness and numbness as initial presentations, mostly with ring enhancement, and brain lesions mostly located in the juxtacortical and periventricular area. In addition, compared with typical MS in Taiwan, our study showed neither more optic and spinal involvement nor a lower frequency of raised IgG indices and OBs in the CSF.
